# Homer

**Published:** 1879-11

**Authors:** 


### HOMES.

Young woman! if a rich young man asks you to marry him, and has no occupation, or trade, or calling, by which he could make a living if he were thrown on his own resources, you may give him your respect, but "give him the mitten."

Whatever may be a young man's qualities, if he is fond, very fond of going to the theater, "refuse" him.

If a young man shows by his conversation that he is an admirer of fast horses, and is pretty well acquainted with the qualities and "time" of the best racing nags of the country, when he asks your hand, "give him the mitten" only.

If you ever hear a young man speak of his father or mother disrespectfully, contemptuously, do not encourage his attentions; he will do the same of you, and in many ways will make your heart ache before you die.

If you know a young man likes to stand around tavern-doors, at the street-corners, and about "groceries," cut your hand off rather than place it in his; he is worth only the "mitten."

If your suitor can tell you a great deal about cards; seems familiar with a multitude of "tricks" which can be performed with the same, and is himself an adept in such things, let him win all the money he may from others, but let him not "win" your heart, for he will "lose it" in a year, and leave you a broken one in its place.

If you know of a "nice young man" who will certainly heir a large estate, who is of a "highly respectable family," who seems to be at home as to the usages, customs, and proprieties of good society, and yet who is indifferent about attending church on the Sabbath-day, who speaks disparagingly of clergymen, who talks about religion in a patronizing way as "a very good thing in its place," particularly for old women, weak young girls and children, never marry him should he ask you.

A shrewd young man, one who is destined to "rise in the world," would never select that girl for a wife who would sit down on a book which chanced to lie on a chair or sofa, who would tread on or over a pocket-handkerchief, or any article of clothing on the floor, rather than stoop to pick it up.

A young lady of taste and refinement would scarcely accept the attentions of a young man the collar of whose coat was usually speckled with dandruff, or whose finger-ends were fringed with black, or whose shirt-bosom was often spotted with tobacco-juice, or was uniformly ruffled or soiled.

Those who are so full of the milk of human kindness as to promise unhesitatingly almost any thing asked of them, and are just as full, later on, of excellent reasons for not having fulfilled these promises, can never make their way into the confidence and respect of the thrifty and the good.

A man of good repute in Wall street, the other day applied to a well-known citizen to rent from him a furnished house. He was refused. A mutual friend expressed surprise. "He stands well on the street." "Yes." "His family are highly esteemed." "Yes." "He is known to be punctual in all his pecuniary engagements." "Yes."

"Well, why won't you let him have your house, at your own price, while you are away?"

"Because he came into my parlor and sat on my sofa with his hat on. Such a man can not have habits of personal neatness. He would spit on my carpets; he would break my chair-backs by tilting them against the wall, and soil it with his unkempt hair. The presumption is, his family are like him; at all events, he alone could injure my furniture more in six months than would be the profits of renting. No, sir! A man who sits in my parlor with his hat on, the first time he ever entered it, can not rent my house at any price."

Each defect in a person's character is read by the observant as easily as the scarlet letter on the back of the erring. Let the young remember that the character will "crop out" in the manners, in the little acts of life, and that, if these are unexceptionable, and if they are uniformly neat, methodical, prompt, and energetic, these qualities will prove a passport to "good places," and to that thrift which brings with it a quiet mind and length of days.

